# Sprint Interval and Sprint Continuous Training Increases Circulating CD34^+^ Cells and Cardio-Respiratory Fitness in Young Healthy Women

**DOI:** 10.1371/journal.pone.0108720

**Published:** 2014-09-29

**Authors:** Emma Harris, Mark Rakobowchuk, Karen M. Birch

**Affiliations:** 1 Multidisciplinary Cardiovascular Research Centre, University of Leeds, Leeds, United Kingdom; 2 School of Sport and Education, Brunel University, Middlesex, United Kingdom; Indiana University, United States of America

## Abstract

**Introduction:**

The improvement of vascular health in the exercising limb can be attained by sprint interval training (SIT). However, the effects on systemic vascular function and on circulating angiogenic cells (CACs) which may contribute to endothelial repair have not been investigated. Additionally, a comparison between SIT and sprint continuous training (SCT) which is less time committing has not been made.

**Methods:**

12 women (22±2 yrs) completed 12 sessions of either SIT (n = 6) or work-matched SCT (n = 6) on 3 days/week. Pre and post-training assessments included brachial artery endothelial function and peripheral blood analysis for CAC number (CD34^+^/CD34^+^CD45^dim^). CAC function was measured by migration and adhesion assays. Cardio-respiratory fitness, carotid arterial stiffness and carotid-radial and brachial-foot pulse wave velocity (PWV) were also evaluated.

**Results:**

CD34^+^ CACs increased following training in both groups but CD34^+^CD45^dim^ did not (Pre CD34^+^: 40±21/10^5^ leukocytes, Post CD34^+^: 56±24/10^5^ leukocytes, main time effect *p*<0.05). Brachial artery flow-mediated dilation (FMD) increased following SIT but SCT had no effect (Pre SIT: 5.0±3.4%, Post SIT: 5.9±3.0%, Pre SCT: 7.2±2.7%, Post SCT: 6.5±2.9%; group x time interaction *p* = 0.08). 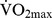 increased in both training groups (Pre: 34.6±4.6 ml•kg•ml^−1^, Post: 36.9±5.4 ml•kg•ml^−1^, main time effect *p*<0.05). CAC function, carotid arterial stiffness and PWV did not change after training (*p*>0.05).

**Discussion:**

SCT involving little time commitment is comparable to SIT in increasing CD34^+^ cell number and 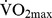. An increased mobilisation of CD34^+^ CACs suggests that sprint training may be an effective method to enhance vascular repair.

## Introduction

Cardiovascular disease (CVD) is the main cause of mortality in the UK, responsible for one third of all deaths [1]. The pathogenesis of atherosclerosis, a key component in the development of CVD, is initiated by endothelial dysfunction [2]. This dysfunction is thought to indicate reduced nitric oxide bioavailability, a factor present in atherosclerotic vessels before vascular structural changes occur [3]. Endothelial dysfunction determined via an impaired brachial artery flow-mediated dilation (FMD) reflects nitric oxide bioavailability [4] and is both indicative of coronary artery endothelial dysfunction [5], [6] and predictive of CVD [7]. Thus, improved endothelial function offers a target for CVD preventative strategies.

A potential method to sustain healthy endothelial function involves the mobilisation of circulating angiogenic cells (CACs). CACs represent a heterogeneous population of haematopoietic stem cells that aid in vascular repair, either through the secretion of angiogenic growth factors [8] or directly by maturation into endothelial cells and incorporation into the endothelium [9]. The mobilisation and function of CACs are impaired in patients with CVD [10] with low numbers of CD34^+^ CAC associated with an increased CVD risk [11]. Improved CAC mobilisation and function [12]–[14] and enhanced endothelial function in central and peripheral arteries can be achieved through exercise [15], [16] thus, exercise training offers a method for endothelial repair and CVD risk reduction.

However, only 6% of men and 4% of women in England meet the recommended guidelines for exercise participation when assessed using accelerometers, with the main barrier to exercise cited as a lack of time [17]. This has led to an increasing number of studies focusing on the benefits of high-intensity training interventions with reduced weekly time commitment. One type of high-intensity exercise emerging within the literature is sprint interval training (SIT), consisting of multiple bursts of maximal exertion separated by rest periods. Few studies have assessed the effects of SIT upon the cardiovascular system, although Rakobowchuk *et al.*, (2008) reported an increased endothelial function and distensibility of the popliteal artery in healthy individuals after only 6 weeks of SIT. A possible mechanism for this improvement is an increased expression of endothelial nitric oxide synthase (eNOS) as observed in rodents following SIT [16]. Additionally, 6 weeks of SIT in sedentary males increased eNOS content in the muscle microvasculature in the exercising limb [18]. However, the effects on systemic endothelial function such as the brachial artery and on CAC number and function have not been investigated following SIT. Additionally, due to the recovery periods involved in SIT sessions, total session duration is not much shorter than the recommended government guidelines of 30 min of moderate-intensity continuous exercise. A less time committing approach would be the performance of a single continuous sprint which would serve to shorten the total training session duration.

Sprint continuous training (SCT) sessions involve one sustained maximal effort sprint without rest periods. This type of training may be more appealing for training purposes than SIT as the time commitment is less. An acute bout of SCT has beneficial effects on metabolic health in overweight/obese men, with immediate increases in insulin sensitivity and reductions in insulin resistance evident [19]. However, the chronic effects of SCT on systemic endothelial function and repair are unknown and have not been compared with SIT. Therefore, the aims of the present study were to determine and compare the effects of work-matched SIT with a less time committing SCT protocol on brachial artery endothelial function, arterial stiffness, cardio-respiratory fitness and CAC number and function. In this first investigation comparing the effects of SIT and SCT on parameters of vascular health, pre-menopausal women will be studied, to determine whether exercise of a high-intensity might alter the vascular phenotype in a young healthy group, likely to undergo vascular changes with ageing.

## Methods

### Ethics statement

Informed written consent and a physical activity readiness questionnaire were completed prior to participation by all participants. The study was approved by the University Of Leeds Faculty Of Biological Sciences Ethics Committee, which conformed to the Declaration of Helsinki.

### Participants

Twelve healthy eumenorrheic females (age: 22±2 yrs; BMI: 23.6±1.8 kg·m^−2^) who were low-moderately active defined as attending between 0–2 structured exercise sessions per week, volunteered for the study. Exclusion criteria included any known cardiovascular, pulmonary or metabolic disease, use of hormonal contraceptives in the last 6 months, pregnancy, smoking, and medication and vitamin use.

### Experimental protocol

Pre-training measurements were undertaken prior to the completion of a 4-week sprint training programme. The exercise training programme duration was chosen to ensure participants were assessed in the same phase of their individual menstrual cycle, and to determine if changes in vascular health can occur as quickly as those reported in skeletal muscle oxidative capacity [20]. For both pre and post-testing assessments, participants attended the laboratory on two separate days. On the first visit the vascular measures were undertaken prior to a fasted venous blood sample collection. Blood was collected in EDTA tubes and separated into two samples for CAC enumeration (1 ml) and functional assessments (30 ml). Participants were instructed not to exercise or consume alcohol in the previous 24 hrs and to refrain from consuming food or caffeine in the 8 to 12 hrs prior to testing. A cardio-respiratory exercise test was completed on the second visit which participants attended in a non-fasted state. Participants were matched for relative maximal oxygen uptake (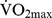) and assigned to either a SIT (n = 6) or SCT (n = 6) training group. Post-testing measures were acquired 4 weeks later between 48 and 72 hrs after the last training session.

### Assessment of brachial artery endothelial function

Assessment of endothelial function was completed in a temperature controlled laboratory (20–24°C) after 20 min of supine rest. An electrocardiograph (ECG) was fitted via a three-lead setup for heart rate (HR) acquisition. Resting HR was calculated as an average over a 5 min period using LabChart software (Labchart 7.0, ADInstruments Pty Ltd, Australia). Brachial artery endothelial-dependent vasodilation was assessed according to established guidelines [21], [22]. A 7 MHz linear array ultrasound probe (Aspen, Acuson; Siemens Medical, Camberley, UK) was used to obtain 20 consecutive ECG gated (end-diastolic) longitudinal images of the brachial artery in the right arm. A 5 min period of forearm ischemia was created by cuff inflation (>50 mmHg above systolic blood pressure; SBP) distal to the ultrasound probe to occlude arterial flow, and 180 end-diastolic images and blood velocity were recorded consecutively from 30 s before cuff release onwards (∼2 min). Images were recorded using vascular imaging software (Vascular Imager, Medical Imaging Applications, Coralville, Iowa, USA) and analysed using semi-automated edge-detection software (Brachial Tools v.5, Medical Imaging Applications) to determine brachial artery diameters. Resting arterial diameter was calculated from an average of 20 consecutive images recorded prior to cuff inflation and peak diameter calculated as the highest value post cuff release from a 3 consecutive cardiac cycle rolling average. Relative FMD was calculated as follows:
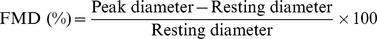
The area under the shear rate curve from cuff release to peak dilation (AUC_peak_) and to 60 s post (AUC_60_) were calculated from 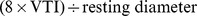, where VTI is the velocity time integral for each period. Peak reactive hyperaemia and shear rate was determined from the highest velocity in the first 10 s post cuff release. FMD was not normalised to shear rate/AUC as not all the assumptions for the use of ratios were met [22].

### Carotid arterial stiffness and structure

Brachial artery blood pressure was measured 3 times (at 5 min intervals) using an automated oscillometric device (Omron M5-I, Omron Healthcare, Europe B.V., The Netherlands) and an average was calculated to determine SBP, diastolic blood pressure (DBP), mean arterial pressure (MAP) and pulse pressure (PP). Carotid arterial stiffness measures were obtained using a combination of ultrasound imaging to determine vessel diameters, and applanation tonometry to estimate carotid artery SBP, as previously described [23]. The ultrasound probe was placed on the right common carotid artery in the longitudinal direction and two video clips were captured for a duration of 20 s at a rate of 15 frames per second (Vascular Imager, Medical Imaging Applications). Immediately post recordings a tonometer (model SPT-301B, Millar Instruments Inc., Texas, USA) was held against the left common carotid artery to capture 20 carotid artery pulse pressure waveforms. These signals were acquired and analysed using a data acquisition system (Powerlab model ML845, ADInstruments) and software (Labchart 7.0, ADInstruments). Video clips were analysed using semi-automated edge-detection software (Carotid Tools Analysis; Medical Imaging Applications) to determine average carotid artery minimum and maximum diameters and end-diastole far-wall intima-media thickness (IMT) from 20 cardiac cycles. End-diastole was identified as the point at which the carotid artery diameter was at a minimum.

As differences in DBP and MAP in different conduit arteries are negligible when in a supine position [24], carotid artery SBP was estimated from an average of 20 carotid artery pulse pressure waveforms (recorded using the tonometer) using linear extrapolation. This equates minimum and mean carotid artery waveform blood pressure values to brachial artery DBP and MAP respectively, and uses maximum carotid artery waveform values as an extrapolation point to estimate carotid artery SBP. Carotid artery PP, cross-sectional compliance (CSC), distensibility and β-stiffness index (SI) were calculated from the following equations [25]:
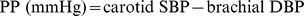


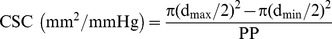


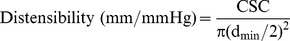


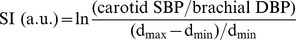
Where d_max_ and d_min_ are the average carotid artery maximum and minimum diameters, respectively.

### Assessment of pulse wave velocity (PWV)

Carotid-radial (PWV_cr_) and brachial-foot pulse wave velocities (PWV_bf_) were measured to assess the effects of SIT and SCT on both peripheral and central arterial stiffness. Given that a strong positive correlation (r = 0.87) exists between PWV_bf_ and aortic PWV [26], PWV_bf_ was used as an estimate of central PWV. Additionally, brachial arterial stiffness is reflected by PWV_cr_ as a measure of peripheral artery stiffness [27], [28], and was therefore used as a measure of upper limb PWV. Pulse pressure waveforms were recorded for 20 s at the carotid, radial, brachial and dorsal pedis arteries using the tonometer described above which was connected to a SphygmoCor PWV system (SCOR-Vx, AtCor Medical Pty Ltd) that enabled simultaneous ECG and pulse pressure waveform recordings. The distance between the sternal notch to these arterial sites were measured in the supine position using a standard tape measure at pre-testing. The distance between the sternal notch and the brachial and radial arteries were measured with the left arm held at a 90° angle to the body. PWV was calculated automatically by the SphygmoCor PWV system by inputting the arterial distances and selecting the intersecting tangents method for determining the pulse transit times (PTT) [29]. The PTT were calculated as the time difference between the peak of the R-wave on the ECG and the foot of the pulse pressure waveforms.

### Enumeration of CACs

**Figure 1:**
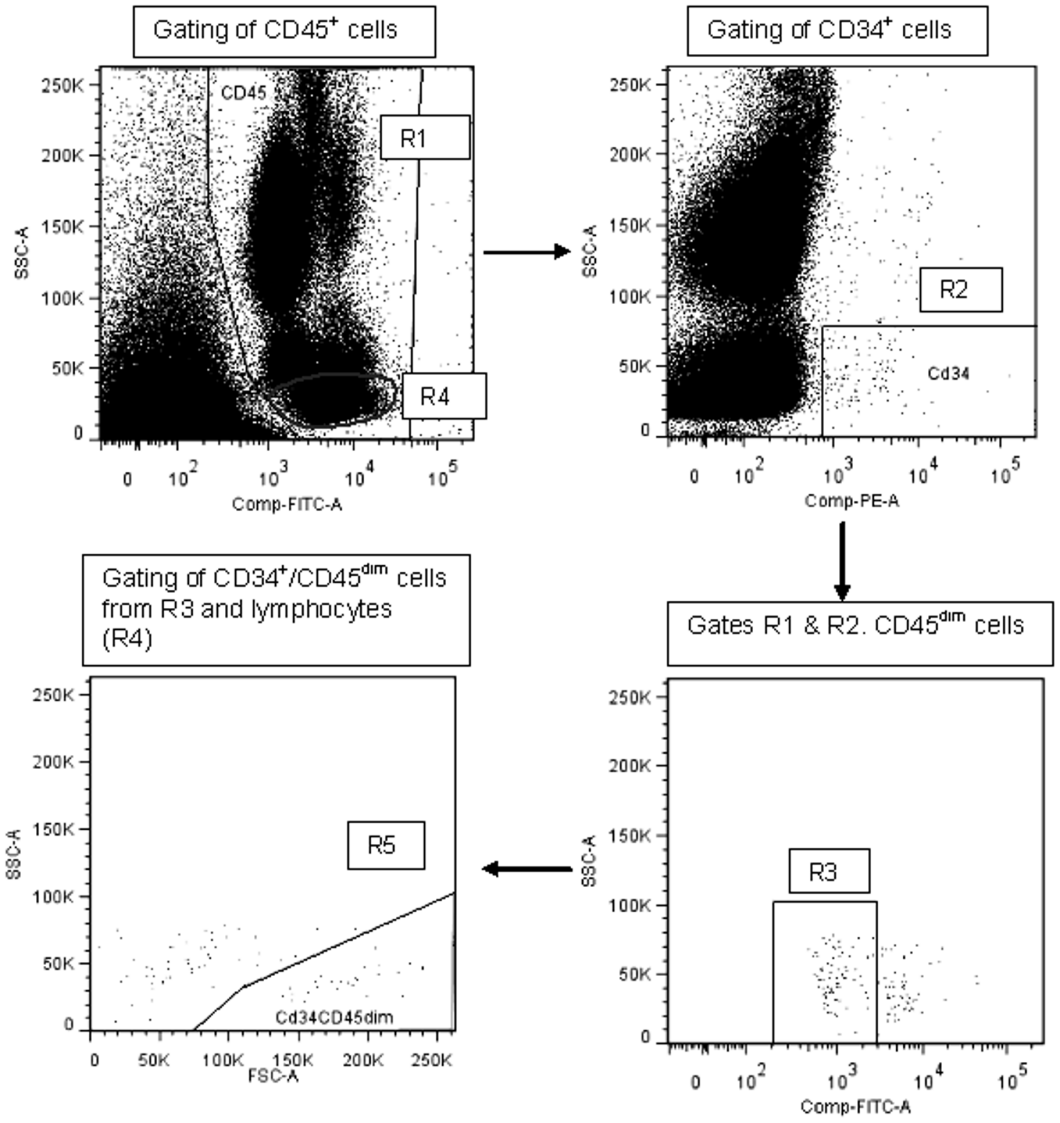
Enumeration of CACs. ISHAGE gating strategy for CAC enumeration. Cells positive for CD45 and therefore leukocytes were gated in R1. Cells in R1 that were positive for CD34 and had low side scatter characteristics were plotted in R2 and defined as CD34^+^ cells. Cells from R2 were gated on a further plot to determine the CD45^dim^ population. Subsequently cells from R3 were back-gated from R4 and gated on a further plot (R5) to define CD34^+^CD45^dim^ cells that are located within the lymphocyte population.

CACs were enumerated using a modified ISHAGE (International Society of Hematotherapy and Graft Engineering) protocol [30]. Briefly, 1 ml of peripheral blood was collected in EDTA tubes and mixed with 1 ml Phosphate buffered saline (PBS). The sample was incubated with 20 µl of Fc-receptor blocker (Miltenyi Biotec, Bergisch Gladbach, Germany) for 10 min, before incubation with fluorochrome-conjugated antibodies, CD34-PE (10 µl) and CD45-FITC (10 µl; Miltenyi Biotec, Bergisch Gladbach, Germany). The sample was lysed, centrifuged at 300 g for 10 min and the pellet resuspended in 1 ml of fluorescence activated cell sorting (FACS) buffer (PBS with 0.5% bovine serum albumin and 0.4% EDTA) before final centrifugation at 300 g for 10 min. The remaining pellet was resuspended in 500 µl of FACS buffer and immediately enumerated by flow cytometry using a FACSCalibur cytometer and CellQuest software (Becton Dickinson, Oxford, UK). Samples were analysed within 2 hrs of blood collection and at least 100,000 events recorded in gate R1 (Figure 1). Cells were enumerated using commercially available analysis software (FlowJo7 6.4). Two subsets of CACs were enumerated as previously described [30]. Cells that were positive for both CD45 and CD34 were termed CD34^+^ cells and measured from gate R5 (Figure 1). CD34+ cells that expressed CD45 at a low intensity (R3, Figure 1), and were found in the lymphocyte region (for exclusion of mature circulating endothelial cells; R4, Figure 1), were termed CD34^+^/CD45^dim^ cells (R5, Figure 1). Isotype controls were not required as other cells and debris (i.e. red blood cells and platelets) that may bind to CD34 antibodies are excluded in R1 [30]. Due to flow cytometer failure, one result at post-testing was not reliable and was excluded from analysis. Cells were expressed as an absolute number per 100,000 leukocytes.

### Functional assessment of CACs

Mononuclear cells were separated from a 30 ml blood sample by ficoll density-gradient centrifugation according to the manufacturer's instructions, (Ficoll Paque PLUS, GE Healthcare) and suspended in endothelial basal medium (EBM) supplemented with 20% fetal calf serum, growth factors and antibiotics (EBM-2, Bullet kit; Lonza, Inc). Cells were plated on 6-well fibronectin coated plates (5×10^6^ per well) and cultured for 7 days at 37°C in 5% CO_2_. On days 2, 4 and 6, non-adherent cells were removed by washing with PBS and fresh medium was added. After 7 days of culture, adherent CACs were detached using trypsin and their adhesive and migratory ability were assessed. At post-testing 1 participant from the SCT group was excluded from the adhesion and migration assays, and 1 participant from the SIT group excluded from the migration assay due to low cell number at harvest.

The ability of CACs to adhere to fibronectin can be used to assess the adhesive ability of CACs *in vitro* [31], [32]. CACs were plated in 24-well fibronectin coated plates (5×10^4^ per well) in 1 ml of supplemented EBM. After incubation for 24 hrs, non-adherent cells were removed by gently washing with PBS and 10 random microscopic images (×200 magnification) were collected for quantification of adherent cells. Assays were performed in triplicate and an average calculated, however in 3 participants at pre-training and 1 participant at post-testing; assays were performed in duplicate due to insufficient cell numbers.

The migratory ability of CACs towards vascular endothelial growth factor (VEGF) can be measured *in vitro* using a Boyden chamber assay [10]. The 8 µm pore Boyden chambers were placed into wells of a 24-well plate containing either 750 µl unsupplemented EBM only (negative control) or combined with VEGF (50 ng/ml). CACs at a density of 4×10^4^ per 500 µl were placed into the Boyden chambers and incubated for 24 hours. Cells were fixed with ethanol and non-migrated cells were removed from the upper surface of the Boyden chamber with a cotton swab. Migrated cells on the lower surface of the chamber were stained with haematoxylin and eosin and 10 random microscopic images (×200 magnification) were collected for quantification of migrated cells. The ability of cells to migrate to a chemoattractant was calculated as the difference between the number of migrated cells to VEGF and the number of migrated cells to unsupplemented EBM. The assay was performed in triplicate in each condition if sufficient cell numbers were available.

### Assessment of cardio-respiratory fitness

Participants performed a seated ramp incremental step exercise test (RISE-105) on an electronically braked cycle ergometer (Excalibur Sport V2.0; Lode BV, Groningen, The Netherlands) for the assessment of 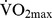 and the lactate threshold (LT). A mouthpiece and nose clip was fitted for breath by breath analyses of pulmonary gas exchange and ventilatory variables (Breeze suite v. 5.0, Medgraphics D-series; Medgraphics, Medical Graphics Corporation, St Paul, MN, USA). Briefly, a 2 min rest period was followed by 2 min of seated cycling at 20 W before initiation of the ramp incremental (RI) test (1 W/4s). The exercise was ended when the participant could no longer maintain a cycling cadence of 50 rpm despite verbal encouragement. The cessation of the RI exercise was followed by a 5 min period of cycling at 20 W before initiation of the step exercise (SE) test at 105% of the work-rate attained at the end of the RI test. The RISE-105 test is an established protocol to confirm that 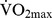 has been achieved rather than 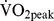 [33]. As previously described, breath-by-breath data were exported and edited in data analysis software (OriginPro 8, OriginLab, Northampton, MA). Breaths were eliminated if 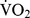 values fell outside four standard deviations around the local mean. A 12-breath rolling average of 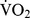 was calculated for both the RI and SE phases of the test, to determine the 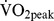 of each phase. A paired t-test determined that the RI and SE 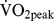 values were not significantly different. Therefore, 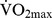 was reported as the average of the 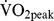 values. The estimated LT was determined using the V-slope method which uses the inflection point of the 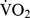 against 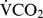 curve as an estimation of LT [34]. This point was further confirmed by a rise in end tidal O_2_ and a plateau in end-tidal CO_2_ [35].

### Exercise training protocol

Participants completed 3 supervised sprint training sessions per week in the laboratory for a 4 week period. All sprints were performed on a cycler ergometer (Ergomedic 874E Peak bike, Monark Exercise AB, Sweden) connected to software (Monark anaerobic test software, Monark Exercise AB HUR OY, Karleby, Finland) for the calculation of average power. In each training session participants in the SIT group completed four 30 s maximal effort sprints (Wingate test) at a resistance equivalent to 7.5% of body weight. The resistance was applied when the participant reached a cadence of 140–150 rpm and each Wingate test was separated by 4.5 min of unloaded pedalling. SIT sessions were based on a previous study [36] whereby vascular function was seen to improve with SIT training.

Participants in the SCT group completed a full SIT session as their first training session for the purpose of calculating the total work achieved from the four 30 s sprints. Total work was calculated in kJ as the sum of the work in each of the 4 Wingate tests from the recording of average power. The total number of revolutions performed on the cycle ergometer to achieve this total work was also recorded. For the remaining training sessions the SCT group completed a single continuous maximal effort sprint. To enable a sustained sprint, the cycle ergometer resistance was reduced by a third of the SIT session resistance to 5% of body mass. Each SCT session was stopped once the participant had completed the equivalent total number of revolutions attained in the 4 Wingate tests in their first SIT session. In this way the SCT and SIT sessions were work-matched. As the resistance was reduced by one third, the total number of revolutions was increased by one third. Thus, both training groups were matched for work relative to their own fitness level. In both groups' sessions, strong verbal motivation was provided to encourage participants to maintain a fast cadence throughout the sprints. All sessions were followed by a short cool-down that was similar between the groups. Thus, the training groups by design both involved maximal exertion sprints, were matched for relative work but differed in regards to session duration and the interval vs. continuous nature of the exercise. Participants in the SCT group completed an extra 30 s Wingate test at the end of training to assess changes in peak power from pre to post-training.

### Statistical analysis

Statistical analysis was performed using statistical software (SPSS version 19.0; IBM Corporation, Somers, NY, USA). Data were assessed for normal distribution using the Kolmogorov-Smirnov test. Pre-testing data were evaluated for training group differences via a Student's independent t-test. The effect of the training interventions was analysed using a mixed model repeated measures ANOVA with time (pre vs. post-training) and training group (SIT vs. SCT) factors. Pearson correlations were performed to establish relationships between variables. Significance was accepted as *p*<0.05 and values were presented as mean ± standard deviation. The between-day reproducibility of relative FMD was calculated from a separate group of 8 young healthy moderately active participants. The participants attended the laboratory on two separate days under the same testing conditions as the trained participants (i.e. fasted, no exercise in the previous 24 hrs). The coefficient of variation was 12.4% and the absolute difference in relative FMD was 0.18%. Given the lack of knowledge on CACs and exercise training in a healthy population, CAC number was selected as the primary outcome. Using the previously reported increase of 8.7 CD34^+^ cells/µl with a standard deviation of 3.0, following a supramaximal bout of exercise in a healthy population [37], a minimum of 8 participants in total were required to obtain 80% power (α = 0.05), in a two-treatment parallel-design study.

## Results

### Participants and training effect

**Figure 2:**
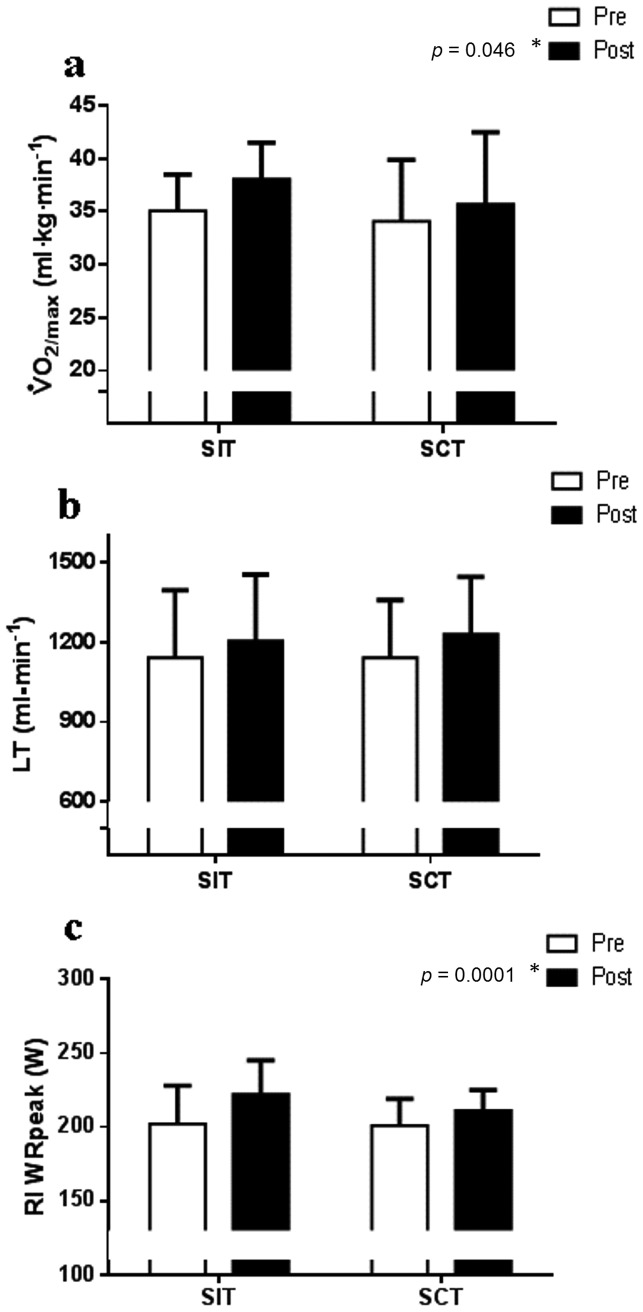
Cardio-respiratory fitness. * indicates a significant pre to post-training difference in both groups (time effect *p*<0.05). a) Relative maximal oxygen uptake (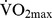) increased following both SIT (n = 6) and SCT (n = 6; main time effect *p* = 0.046) with no group x time interaction (*p* = 0.49). b) The estimated lactate threshold (LT) followed a trend to increase in both training groups (main time effect *p* = 0.08) with no group x time interaction (*p* = 0.30). c) The ramp incremental (RI) test work-rate peak (WRpeak) significantly increased in both groups (main time effect *p* = 0.0001) with a greater increase following SIT (group x time interaction *p* = 0.05).

Participant characteristics are displayed in Table 1. There were no pre-training differences present between the training groups in any variable (*p*>0.05). BMI, resting HR and brachial artery BP did not change following training (*p*>0.05). By design both the training groups' session duration (SIT: 20 min, SCT: 3.5±0.2 min) and resistance applied to the cycle ergometer (SIT: 5.1±0.6 kg, SCT: 3.4±0.4 kg) were greater in the SIT group. Work completed per session did not significantly differ between the groups (SIT: 45.9±6.3 kJ vs. SCT: 47.6±6.2 kJ). Peak and average power calculated for a single 30 s Wingate test did not increase from session 1 to post-training (*p*>0.05). Both absolute (*p* = 0.048) and relative 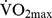 (*p* = 0.046) increased with training with no interaction between group x time (*p*>0.05, Table 1, Figure 2a). The estimated LT also increased with training in both groups (Figure 2b); although significance was not reached (*p* = 0.08) the 95% confidence interval (CI) for pre to post-training difference ranged from −11 to 164 ml/min. The RI test duration and work-rate achieved at the end of the RI stage (WRpeak) significantly increased following training in both groups (*p*<0.001) with a greater increase following SIT (group x time interaction for both *p* = 0.05; Table 1, Figure 2c).

**Table 1:** Participant characteristics at pre and post 4 weeks of either sprint interval (SIT) or sprint continuous training (SCT).

	SIT (n = 6)		SCT (n = 6)	
	PRE	POST	PRE	POST
Body mass index (kg·m^−2^)	23.6±1.8	23.8±1.6	23.1±2.3	22.9±2.6
Resting heart rate (bpm)	55±10	56±6	62±8	64±12
Brachial artery SBP	115±7	117±11	112±12	111±11
Brachial artery DBP	73±5	72±11	76±9	72±7
Brachial artery MAP	87±5	87±10	88±10	85±8
Absolute 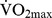 (L·min^−^1)*	2.34±0.37	2.55±0.31	2.24±0.22	2.30±0.21
RI test duration (min)*	12.14±1.74	13.44±1.55	12.06±1.18	12.73±0.9
Lactate threshold (%)	48.4±7.4	46.4±5.4	49.0±6.4	51.9±8.1
Peak reactive hyperaemia (cm·s^−1^)	98.8±23.6	97.3±19.4	82.0±14.7	90.1±8.7
Peak shear rate (s^−1^)	2629±1064	2564±803	2126±449	2395±301
AUC_peak_ (a.u.)	31325±11174	30406±13875	27646±6595	31396±6366
AUC_60_ (a.u.)	39815±14654	39648±16146	39596±5261	43901±6012
Insonation angle (°)	69±1	68±2	68±2	68±1
Brachial artery baseline diameter (mm)	3.2±0.8	3.2±0.8	3.1±0.5	3.1±0.5
Absolute FMD (mm)	0.15±0.09	0.18±0.07	0.22±0.08	0.20±0.08
Time from cuff release to peak diameter (s)	38±8	35±4	34±9	34±6
CAC adhesion per microscopic image (SIT: n = 6; SCT: n = 5)	11±8	8±6	13±8	11±12
CAC migration per 10 microscopic images (SIT: n = 5; SCT: n = 5)	2±3	4±7	1±4	2±2

* indicates a significant main time effect (*p*<0.05).

No group differences at baseline or group x time interactions were observed (*p*>0.05). Absolute FMD group x time interaction was close to significant (*p* = 0.08). RI test duration group x time interaction was close to significant (*p* = 0.05). Bpm = beats per minute, SBP = systolic blood pressure, DBP = diastolic blood pressure, MAP = mean arterial pressure, 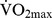 = maximal oxygen uptake, RI = ramp incremental, AUC = area under the shear rate curve, FMD = flow-mediated dilation and CAC = circulating angiogenic cell.

### Brachial artery endothelial function

**Figure 3:**
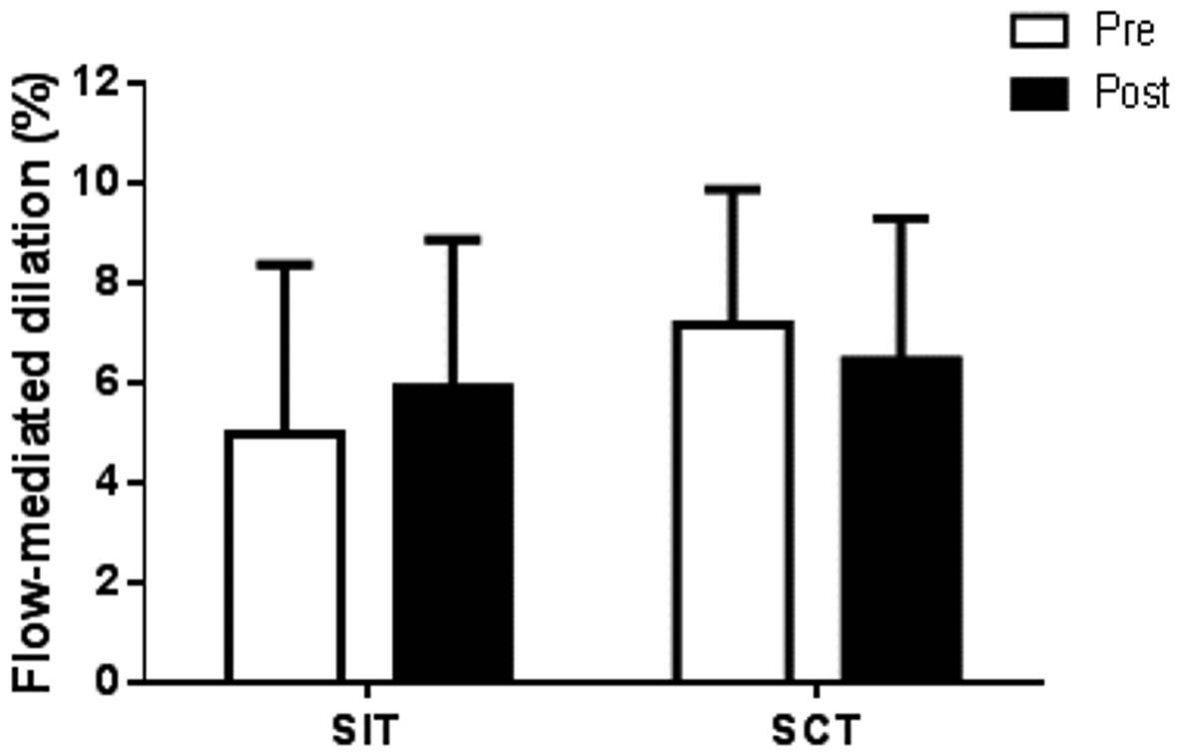
Brachial artery endothelial function. Brachial artery FMD displayed a trend for an increase following SIT (n = 6) but no change following SCT (n = 6; main time effect *p* = 0.81; group x time interaction *p* = 0.08).

Resting brachial artery diameter and time from cuff release to peak dilation were unchanged following training in both groups (*p*>0.05, Table 1). However, absolute and relative FMD showed a trend for an increase (FMD_rel_ by ∼19%, FMD_abs_ by ∼23%) following SIT but no change following SCT (Table 1, Figure 3). Although the main time effect was not significant (*p* = 0.81), the group x time interaction showed a trend (*p* = 0.08), with 67% of participants in the SIT increasing FMD (FMD_rel_: 95% CI for pre to post-training difference: −0.59 to 2.43%) and 67% of SCT participants exhibiting reduced FMD (FMD_rel_: 95% CI for pre to post-training difference: −2.29 to 0.86%). Larger increases in absolute FMD occurred in participants with lower pre-training levels of absolute FMD (r = −0.57, *p* = 0.06). Peak reactive hyperaemia, peak shear rate, AUC_peak_ and AUC_60_ did not change in either training groups (*p*>0.05, Table 1).

### Arterial stiffness

Carotid to radial and brachial to foot PWV did not alter following training in either group (*p*>0.05) indicating that sprint training did not affect upper limb or central PWV (Table 2). Carotid artery IMT, distensibility, cross-sectional compliance and stiffness index were also unaltered following both types of training (*p*>0.05; Table 2).

**Table 2:** Arterial stiffness pre and post either sprint interval (SIT) or sprint continuous training (SCT).

	SIT (n = 6)		SCT (n = 6)	
	PRE	POST	PRE	POST
PWV_cr_ (m·s^−1^)	6.0±0.8	6.2±0.5	6.6±0.8	7.4±0.7
PWV_bf_ (m·s^−1^)	7.4±0.9	7.8±1.4	8.2±1.6	7.4±1.1
Carotid artery IMT (mm)	0.31±0.10	0.36±0.07	0.33±0.09	0.35±0.06
Carotid artery PP (mmHg)	29±4	31±5	25±3	27±5
Carotid ΔCSA within heart cycle (mm^2^)	6.2±1.3	6.0±0.7	6.2±1.5	6.2±1.5
Carotid artery CSC (mm^2^/mmHg)	0.22±0.05	0.19±0.03	0.25±0.06	0.24±0.08
Carotid artery DD (mm/mmHg)	0.01±0.002	0.01±0.002	0.01±0.002	0.01±0.002
Carotid artery SI (a.u.)	3.3±0.8	3.6±0.8	3.0±0.6	3.4±1.2

No group differences at pre-training, training effects or group x time interactions were found (*p*>0.05). PWV_cr_ = carotid-radial pulse wave velocity, PWV_bf_ = brachial-foot pulse wave velocity, IMT = intima-media thickness, PP = pulse pressure, CSA = cross-sectional area, CSC = cross-sectional compliance, DD = distensibilty, SI = β-stiffness index.

### Training effect on CAC number

**Figure 4:**
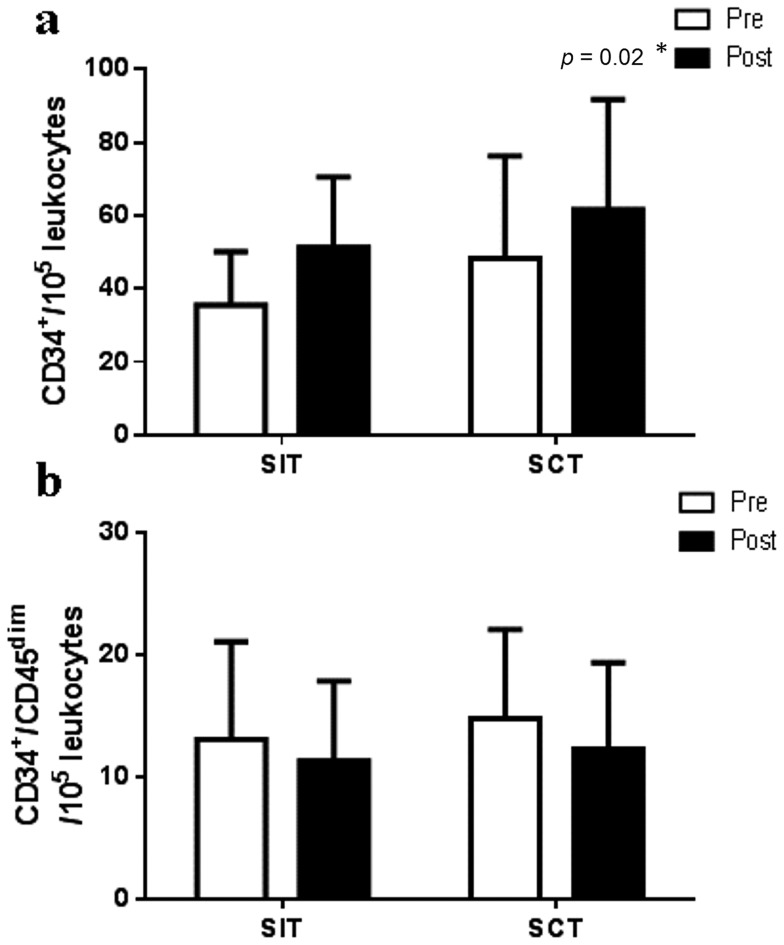
Circulating angiogenic cells. * indicates a significant pre to post-training difference in both groups (time effect *p*<0.05). a) CD34^+^ cells increased following both SIT (n = 6) and SCT (n = 5; main time effect *p* = 0.02) with no group x time interaction (*p* = 0.83). However, b) CD34^+^/CD45^dim^ cells did not change following either type of training (main time effect *p* = 0.21; group x time interaction *p* = 0.67).

Circulating CD34^+^ cells increased following both SIT (by ∼44%; n = 6) and SCT (by ∼28%; n = 5; main time effect *p* = 0.02) with no group x time interaction (*p* = 0.83; Figure 4a). However, CD34^+^/CD45^dim^ did not change following either type of training (main time effect *p* = 0.21, group x time interaction *p* = 0.67; Figure 4b). Changes in CD34^+^ cells with training did not correlate with pre-training or changes in FMD (Pre FMD r = 0.14, pre to post-training delta FMD r = 0.34, *p*>0.05).

### CAC function

The function of CACs did not significantly improve with either type of training (main time effect, adhesion *p* = 0.47, migration *p* = 0.63; group x time interaction *p*>0.05; Table 1). However, whilst training induced changes in CAC adhesion were not related to pre-training levels (*p*>0.05), changes in CAC migration were greater in participants with lower pre-training levels of CAC migration (r = −0.67, *p* = 0.03).

## Discussion

The present study to our knowledge is the first to examine the effects of sprint training on brachial artery function as an indicator of systemic vascular function, and the mobilisation and function of circulating cells that may contribute to endothelial repair. Furthermore, novel comparisons were made between SIT and SCT to explore whether sprint training involving a continuous work-rate stimulus has differential effects on the vasculature than work-matched sprinting of an interval nature. The main findings were that despite the lower training time commitment, SCT improved cardio-respiratory fitness to a similar extent as SIT. Furthermore, increased mobilisation of circulating CD34^+^ cells was observed following both types of training, but arterial stiffness, CD34^+^/CD45^dim^ mobilisation and CAC function remained unchanged. Additionally, there was a trend in brachial artery FMD to increase following SIT but no change following SCT.

### Improvements in exercise tolerance

Recently, a “lack of time” has been highlighted as the general publics' main barrier to exercise thus, several studies have focused on how supramaximal but less time committing exercise can be equally as beneficial to health and fitness as longer methods of exercise training. Improvements in 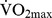 by ∼10% have been observed following as little as 6 weeks of Wingate test based SIT [38], and the current study has observed similar changes. Additionally, following both training types, there was a near significant improvement in the LT and a significant increase in the RI test duration and WR_peak_, with greater improvements following SIT. Since 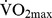 is a strong predictor of future cardiac events [39], and the LT and WR_peak_ are markers of exercise tolerance that are associated with poorer cardiac outcomes [40], this type of training may be favourable to those who wish to rapidly increase their cardio-respiratory fitness and/or cardiovascular health. However, given that our results are in a population of young healthy females, further studies are required to investigate whether these findings can be translated to populations that have poor cardiovascular health.

### Differential effects of SIT and SCT on brachial artery endothelial function

Our results indicated a close to significant trend for an increase in brachial artery FMD following SIT but no change after SCT, without any changes in peak reactive hyperaemia, peak shear rate, AUC_60_ or AUC_peak_. Given that brachial artery FMD is largely nitric oxide dependent [4], this result suggests that SIT provides a greater stimulus for increased nitric oxide bioavailability than SCT. Improvements in brachial artery endothelial function following lower limb exercise training in healthy populations have occurred as early as 2 weeks and begun to return to baseline at 4 weeks due to arterial remodelling [41]. This may explain why other studies assessing upper limb endothelial function in healthy young participants following longer training protocols have not observed a change [42]. Therefore, it is plausible that if brachial artery endothelial function in the present study had been assessed after 2 weeks, a larger magnitude of change may have been present.

Increases in brachial artery FMD following exercise training are believed to be caused by increases in brachial artery blood flow antegrade shear stress during exercise sessions [43], which induce nitric oxide release through activation of eNOS [15], [44]. However, in the initial 5 min following lower limb cycling onset, mean brachial artery blood flow decreases due to an increase in retrograde flow caused by forearm resistance vessel vasoconstriction [45], [46]. As opposed to antegrade shear stress, retrograde shear stress is pro-atherogenic as it increases reactive oxygen species and the expression of adhesion molecules which reduce nitric oxide bioavailability [47]. Additionally, greater oscillatory and retrograde shear rate in the brachial artery, activates the endothelium and induces damage, evidenced by significantly higher levels of circulating endothelial microparticles during and acutely after a period of induced disturbed flow [48]. Furthermore, retrograde shear stress reduces brachial artery FMD in a dose-dependent manner [49]. Since SCT duration was less than 5 min in our study, it is possible that the brachial artery endothelium experienced elevated retrograde shear stress unopposed by increases in antegrade shear stress, which may have negated improvements in endothelial function by reducing nitric oxide bioavailability. In contrast, SIT duration was 20 min which allowed sufficient time for antegrade shear stress to increase and for retrograde shear stress to return to baseline due to reductions in downstream peripheral resistance caused by thermoregulatory cutaneous vasodilation [46]. Thus, SIT may be more beneficial for brachial artery adaptations due to increases in antegrade shear stress that augment nitric oxide production, whereas SCT duration may be too short to induce the increases in antegrade shear stress required for improvements in FMD. However, further studies investigating blood flow responses to SIT and SCT are required to validate this suggestion.

Superior health benefits have been reported following interval exercise training compared to continuous exercise training, which have often been related to a higher exercise intensity, shear stress and work done experienced during interval exercise [50]. In the present study, SCT and SIT were both of a high intensity and matched for work. Therefore, it may be that the profile of the repeated increments and decrements in work-rate provided a greater stimulus for vascular adaptations than a continuous work-rate. Evidence from *in vitro* studies on endothelial cells suggests that the temporal gradients in shear stress are more important than the magnitude of the shear for eNOS activation and nitric oxide production. A rapid increase in shear stress from a pre-existing level creates a burst in nitric oxide production [51]. Subsequently, exposure to a sustained magnitude of this shear stress reduced the rate of nitric oxide production. Additionally, repeated impulses in shear stress have been seen to produce a large increase in nitric oxide production, whereas a slow ramp increase in flow to the equivalent magnitude had no effect [52]. This was thought to be due to the activation of eNOS by platelet endothelial cell activation molecule-1 (PECAM-1) which may only sense shear stress when rapid changes occur due to its sheltered position at cell-cell junctions [52]. Therefore, given that our SIT sessions involved 4 periods of rapid increases in shear stress compared to a single increase in the SCT, it seems reasonable to suggest that SIT provided a greater stimulus for nitric oxide synthesis. Furthermore, it is likely that the elevation in shear stress would have occurred for longer during SIT due to a longer session duration. The measurement of shear stress during SIT and SCT and further *in vitro* studies examining endothelial cell responses to continuous and periodic fluctuations in shear stress are required to validate these suggestions.

### Unaltered arterial stiffness following sprint training

PWV, a measure of arterial stiffness associated with increased CVD risk [24], did not change with training both centrally and in the upper limb, indicating an unaltered arterial stiffness. Conversely, a previous study has observed an increased popliteal arterial distensibility following 6 weeks of SIT [36]. This suggests that sprint training can reduce arterial stiffness in the exercising limbs but not centrally or in the untrained upper limbs in a healthy population.

Carotid arterial distensibility and IMT are linked to CVD progression [53]. In the present study, carotid arterial stiffness and IMT did not change following SIT or SCT, in agreement with a previous study following SIT in healthy individuals [36]. Healthy pre-training vasculature may explain the lack of a training effect, as carotid distensibility and IMT are similar to previously reported values in a healthy population [36], [54].

### Training effect on CAC mobilisation and function

The present study is the first to evaluate the effects of sprint training on CAC mobilisation and function and demonstrated an increase in circulating CD34^+^ cells following SIT and SCT but no change in its subpopulation CD34^+^/CD45^dim^, suggesting that a different subpopulation of CD34^+^ cells were mobilised. The enhanced CD34^+^ cells did not correlate with FMD at pre-training or training induced changes in FMD, indicating that these cells may not have been directly involved in the increase in brachial artery endothelial function. It is possible that the elevated numbers of CD34^+^ cells were recruited to other parts of the body such as the arteries supplying blood to the exercising muscles. Furthermore, cultured CACs adhesive and migratory ability did not alter following both training programmes. Conversely, increases in CAC function following exercise training have been documented in populations with or at risk of CVD [12], [13], [55]. However, healthy individuals do not exhibit impaired CAC function [10], which likely explains why an increase in CAC function was not observed in the present study. Furthermore, increases in CAC migratory ability occurred in those with lower pre-training levels in the present study, supporting the suggestion that exercise training increases CAC function, only when an impairment in CAC function is present at pre-training.

CD34^+^ haematopoietic cells have been reported to migrate towards arterial injury [32], adhere to implanted grafts [56] and restore circulation to the ischemic limb of mice [57], providing evidence for their role in endothelial repair and angiogenesis. The present study is the first to show elevated numbers of CACs following exercise training in a healthy population and suggests an increased reparative potential if required. In contrast, no change in CACs was observed following 8 weeks of continuous endurance training in healthy older men [58] and in our lab following 6 weeks of moderate intensity interval training in healthy young adults [42]. Although in the latter study, some participants exhibited a sustained mobilisation of CACs following heavy intensity interval training, which may have been a result of the higher exercise intensity. This evidence combined with the sustained mobilisation of CACs following both SIT and SCT in the present study, suggests that the high intensity nature and not the duration or the interval vs. continuous nature of the exercise, was the main contributor to increased CACs. This supports the theory that a greater metabolic stress during exercise leads to a sustained upregulation of CACs in healthy individuals, as both training programmes in the present study involved maximal exertion sprints that have been shown to achieve 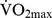 (unpublished data from our laboratory). With increases in exercise intensity, greater levels of oxidative stress are produced [59], which activates the endothelium, leading to secretion of vascular endothelial growth factor and stromal cell-derived factor-1 alpha from endothelial cells that aid in the mobilisation and homing of CACs [60]. Conversely, acute bouts of moderate intensity exercise below the LT in healthy individuals have been shown to elevate CACs [14], [61] via a nitric oxide mediated pathway, with levels returning to baseline after 24 hrs. Taken together, these data suggest that for chronic increases in CACs following exercise training in healthy individuals, a higher level of oxidative and metabolic stress is required during exercise sessions.

### Study limitations

Comparisons between studies examining exercise induced CAC mobilisation are difficult due to the different antigens used to define cells and the various methods and gating strategies used for cell enumeration. This may explain why previous studies have shown no change in CAC number following exercise training in healthy individuals, whereas the present study reported an elevation. We chose to examine CD34^+^ cells since out of all its subpopulations, CD34^+^ cells were the best predictor of CVD [11]. We observed no alteration in CAC function following training, however, the paracrine function of these cells was not assessed. Addition of this measurement may have given insight into the impact the increase in CD34^+^ cells had on the vasculature.

The population studied were young, healthy and free from cardiovascular disease. Therefore, further studies are required to adapt this type of training to make it appropriate for individuals with or at risk of CVD. Additionally, sprint training requires motivation and specialised equipment that is not accessible to the public. Thus, future work should focus on how this type of training can be made applicable for the general public. A recent study has started progress by adapting SIT for patients with coronary artery disease, and found increases in brachial artery FMD [62]. Training sessions were 2/week for 12 weeks and involved cycling at 90% of maximal HR for 60 s with a 60 s recovery period, repeated 10 times. However, other measures of vascular health such as arterial stiffness and CAC number and function following this type of training in CVD at risk populations have not been studied. Finally, the duration of the training programme was relatively short (4 weeks); therefore, it is unknown whether greater increases in the parameters measured may occur with further training.

## Conclusions

Sprint continuous training elicits similar increases in cardio-respiratory fitness and stem cell mobilisation as work-matched sprint interval training in a young healthy population, even though on average SCT session duration was only 3.5 min. However, brachial artery endothelial-dependent FMD may benefit more so following SIT due to the type or profile of shear stress experienced during the exercise. Increases in these variables in CVD populations may be of great benefit as these individuals exhibit impaired vascular function. Therefore, future studies are warranted to adapt this type of training and determine whether those with or at risk of CVD can gain the same benefits as a healthy population.
